# Case report: Outflow reconstruction with pre-frozen allograft blood vessels during *in vivo* partial hepatectomy followed by *ex vivo* tumor resection and partial liver autotransplantation for locally advanced hepatocellular carcinoma with background of cirrhosis

**DOI:** 10.3389/fonc.2024.1432274

**Published:** 2024-12-13

**Authors:** Jun-Ze Chen, Cheng Zhang, Rui-Ling Su, Yong-Yuan Jian, Kai-Yong Huang, Xue-Lin Tan, Zhao Chen, Yong-Xin Liao, Chun-Qiang Dong, Kun Dong

**Affiliations:** Department of Organ Transplantation, the First Affiliated Hospital of Guangxi Medical University, Nanning, China

**Keywords:** autotransplantation, hepatocellular carcinoma, hepatectomy, *in vivo*, *ex vivo*, vascular allograft

## Abstract

*Ex vivo* surgery and autotransplantation may provide a promising option for radical resection of conventionally unresectable liver tumors. Two cirrhotic patients with hepatocellular carcinoma (HCC), which has an “awkward seat” located in the “intrahepatic vascular triangle area (IVTA)” that consists of the middle hepatic vein (MHV), the right branches of the Glisson sheath, and the inferior vena cava (IVC), underwent *in vivo* extended right-half hepatectomy followed by *ex vivo* tumor resection and partial liver autotransplantation. Innovatively, the outflow of the tumor-free liver was reconstructed *ex vivo* using pre-frozen allograft blood vessels from brain-dead donors; the patients recovered well postoperation. We report the surgical experience to provide a novel curable surgical procedure for locally advanced IVTA liver tumors.

## 1 Introduction

Since *ex situ* hepatectomy was first reported by Rudolf Pichlmayr in 1988 (1), the combination of *ex vivo* hepatectomy and autotransplantation technique was introduced to overcome the barriers of poor intraoperative exposure, mass blood loss, insufficient residual liver volume, narrow surgical margins, or non-anatomical hepatectomy for both conventionally unresectable malignant and benign lesions in the liver (2). The modified technique of *in vivo* partial hepatectomy followed by *ex vivo* tumor resection and partial liver autotransplantation may be a promising option for cirrhotic patients to avoid as much as possible the high risk of severe liver damage or even liver failure (3). To date, this technique has only been reported in about six patients with hepatic alveolar echinococcosis or biliary tract cancer, and the blood vessel reconstruction was conducted with artificial blood vessels (3). In the present study, we report two cirrhotic patients with intrahepatic vascular triangle area (IVTA) hepatocellular carcinoma (HCC) who received *in vivo* right-half hepatectomy followed by *ex vivo* tumor resection and partial tumor-free liver autotransplantation. Innovatively, the outflow of the residual tumor-free liver was reconstructed *ex vivo* with pre-frozen allograft blood vessels from brain-dead donors. Surgical skills were analyzed, and postoperative prognoses were assessed.

## 2 Case report

### 2.1 Case 1

A 54-year-old male patient was referred to our hospital for a liver mass detected by computed tomography (CT) during a routine physical examination, accompanied by an occasional sensation of fullness from 20 days before his admission. Except for a 3-year history of hepatitis B virus (HBV) infection, the patient did not report any diseases or hospitalizations in his past medical history; he reported diseases in his family history. No abnormal finding was detected through physical examination. Laboratory results showed he was positive for alpha-fetoprotein (AFP) and did not have enough residual liver volume of 363 mL [graft-to-recipient body weight (GRBW) = 0.59%] if conventional right hepatectomy was performed.

### 2.2 Case 2

A 39-year-old male patient suffered from discomfort in the upper abdomen and weakness for 18 days. After the liver mass was detected by magnetic resonance imaging (MRI), he was admitted to the hospital. His medical history did not include any positive pathology except for a 3-year history of HBV infection. Laboratory results showed he was negative for AFP and did not have enough residual liver volume of 360 mL (GRBW = 0.58%) if conventional right hepatectomy was performed.

**Figure 1:**
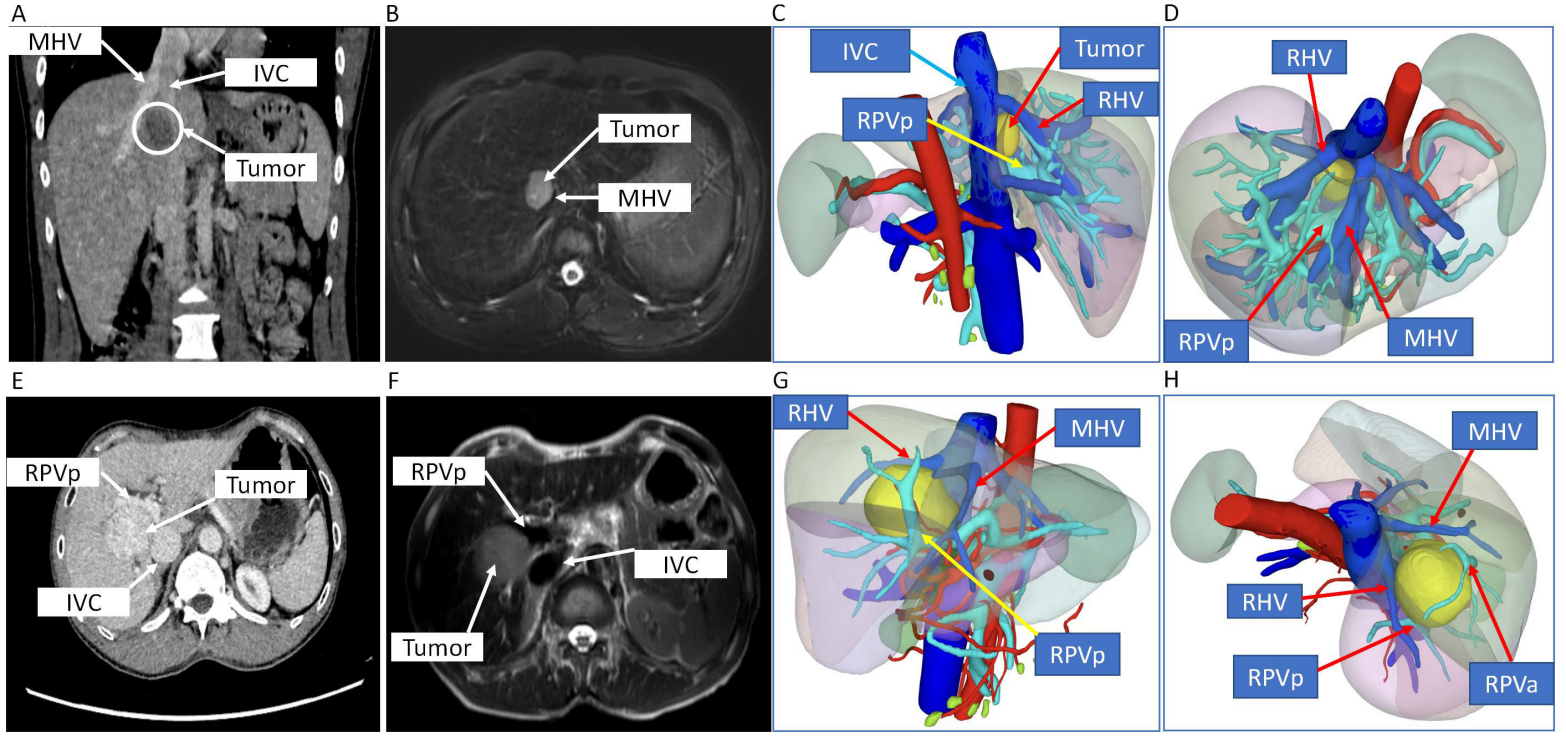
CT scan, MRI, and 3D digital modeling of liver imaging of the two patients. **(A–D)** Imaging examination of case 1. **(A)** Coronal position of CT scan of the tumor in the liver. **(B)** T2-weighted image of MRI of the tumor in the liver. **(C, D)** 3D digital modeling of liver and tumor. **(E–H)** Imaging examination of case 2. **(E)** Transverse position of CT scan of the tumor in the liver. **(F)** T2-weighted image of MRI of the tumor in the liver. **(G, H)** 3D digital modeling of liver and tumor. MHV, middle hepatic vein; IVC, inferior vena cava; RHV, right hepatic vein; RPVa, right anterior portal vein; RPVp, right posterior portal vein.

Abnormal or positive laboratory results of the two patients including blood biochemical examination and imaging examination are shown in **Supplementary Table S1** and **Figure 1**.

CT scan, MRI, and 3D digital modeling of liver imaging of the two patients showed that the tumors were located in S8 or S5/8 and typical IVTA HCC (**Figure 1**).

### 2.3 Surgical procedure

**Figure 2:**
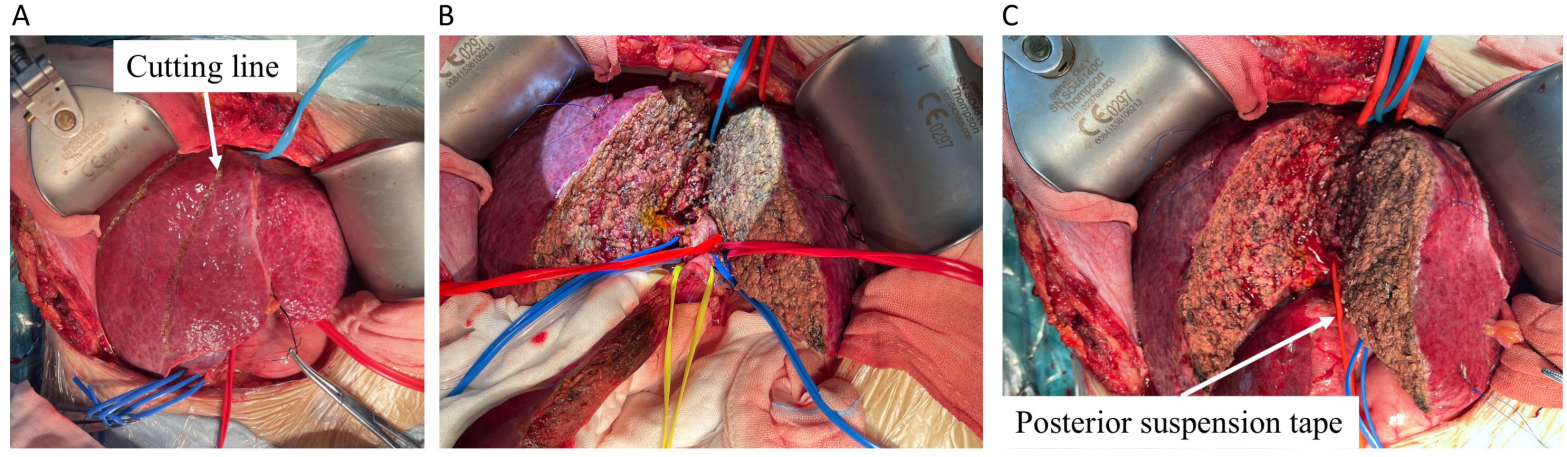
The split of half liver containing tumor. **(A)** The liver was transected using CUSA marked by cutting line (white arrow). **(B)** The hepatoduodenal ligament and hilar plate were dissected, and the main branches of the hepatic vein (blue tape), hepatic duct (yellow tape), portal vein (blue tape), and hepatic artery (red tape) were dissected to expose and hang the suspension straps in different colors. **(C)** The posterior portion of the residual liver was separated from the IVC by a red elastic urinary catheter and then completely transected. CUSA, cavitron ultrasonic surgical aspirator.

A right subcostal reverse “L”-shaped incision with midline extension was applied to enter the abdomen and expose the liver. No signs of metastatic lesions were found around the liver and the adjacent tissues or organs. A nodular liver without obvious tumor involvement to parenchyma was observed. After releasing the falciform and coronary ligaments of the liver, intraoperative ultrasonography was conducted to confirm the tumor margins, a line on the liver surface was cut, and any potential hepatic lesions were ruled out (**Figure 2A**). Then, the whole liver was released by dissecting the ligaments around the liver, exposing the hepatic veins. The hepatoduodenal ligament and hilar plate were dissected to expose and hang the main branches of the hepatic duct, portal vein, and hepatic artery, especially those belonging to the part of the liver would be removed (**Figure 2B**). After the supra- and infra-hepatic inferior vena cava (IVC) was completely dissected for suitable placement of clamps, cavitron ultrasonic surgical aspirator (CUSA) was used to transect the liver parenchyma along the pre-setting cutting line (**Figure 2C**). The final incision margins of the tumor were adjusted by intraoperative ultrasonography in real-time to complete the *in vivo* extended right-half hepatectomy.

**Figure 3:**
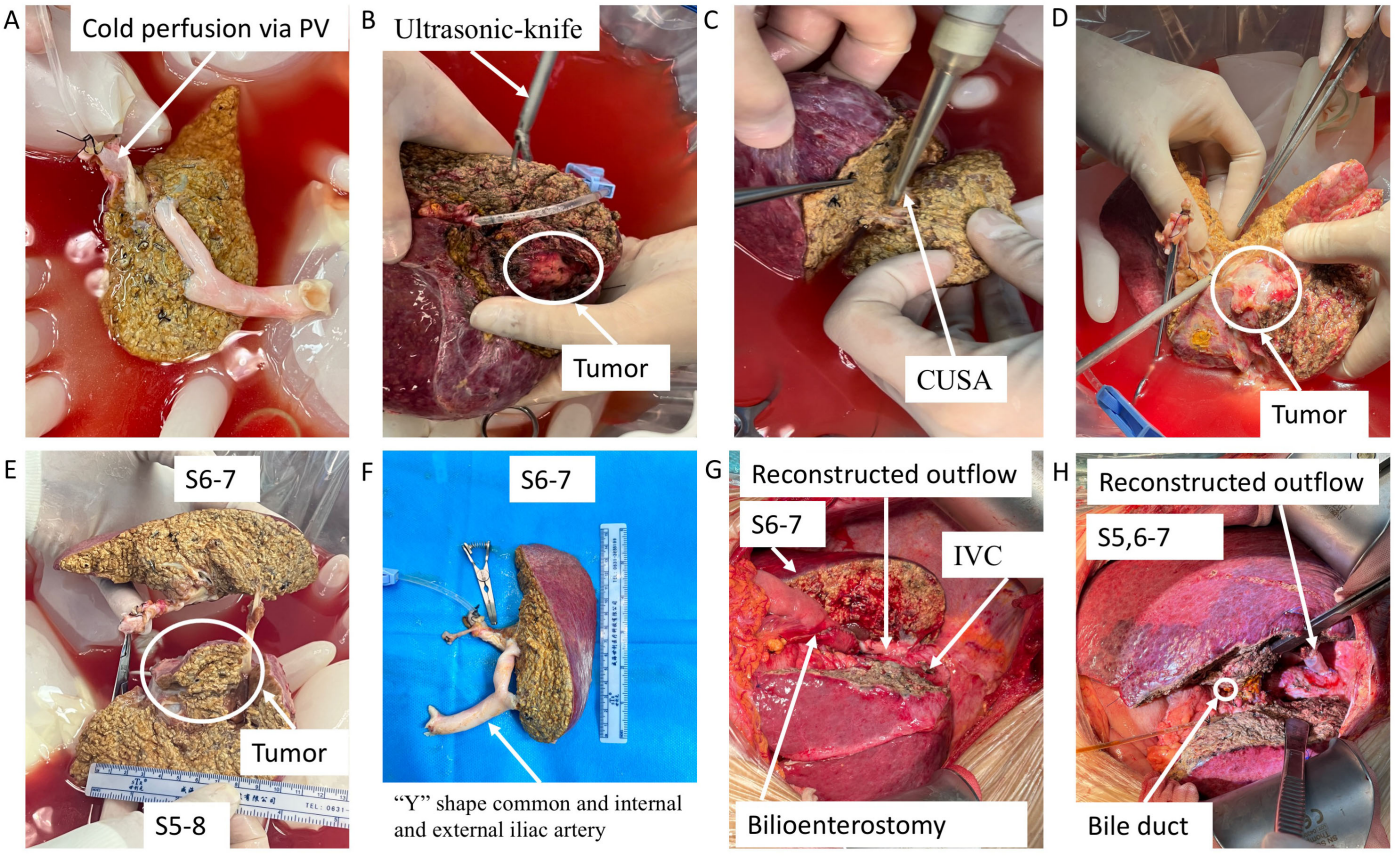
Key surgical steps of *ex vivo* tumor resection and partial liver autotransplantation and outflow reconstruction with pre-frozen allograft blood vessels. **(A)** The excised liver containing tumor was preserved in pre-cooling ice basin and flushed with HTK solution. **(B–E)** The tumor was completely removed *ex vivo* using CUSA, ultrasonic knife, and intraoperative ultrasonography. **(F)** The portal vein and outflow of the remaining liver segments were reconstructed using pre-frozen allograft vessels. **(G, H)** The tumor-free liver was autotransplanted into the anatomical position. PV, portal vein; HTK, histidine–tryptophan–ketoglutarate; CUSA, cavitron ultrasonic surgical aspirator.

After reserving appropriate anastomotic length, the artery, bile duct, portal vein, and right and middle hepatic veins of the right half liver were blocked and cut; then, the right half liver was removed, placed into a pre-cooling ice basin, and flushed with histidine–tryptophan–ketoglutarate (HTK) solution via portal vein and hepatic artery to clear the residual blood immediately (**Figures 3A, B**). With CUSA, ultrasonic knife, and intraoperative ultrasonography, the tumor was completely removed *ex vivo*; the artery, bile duct, and portal vein were dissected along the margins of the tumor with scissors and skeletonized to avoid residual tumor tissue. However, the main part of the bile duct and portal vein very closely adhered to the tumor to be dissected, so they were cut off. Then, the V and VIII liver segments containing tumors were transected and removed as a whole, while the main trunks of the right and middle hepatic veins were also removed together (**Figures 3C–F**). The portal vein and outflow of the remaining VI and VII liver segments were reconstructed using pre-frozen allograft vessels (within 1 month at ~−80°C) of vena cava inferior using a “Y”-shape common and internal and external iliac arteries from brain-dead donors (**Figures 3A, F**), respectively. Then, the tumor-free liver was moved back to the anatomical position, and the reconstructed outflow tract, portal vein, hepatic artery and were anastomosed end-to-end using 5/0 or 7/0 prolene, while a biliary enterostomy by end-to-side was performed for case 1 (**Figures 3G, H**). The blood flow spectrum of all the reconstructed vessels was confirmed by ultrasound after anastomosis; then, the abdomen was closed, and the patients were monitored in the intensive care unit (ICU) on the third day.

**Figure 4:**
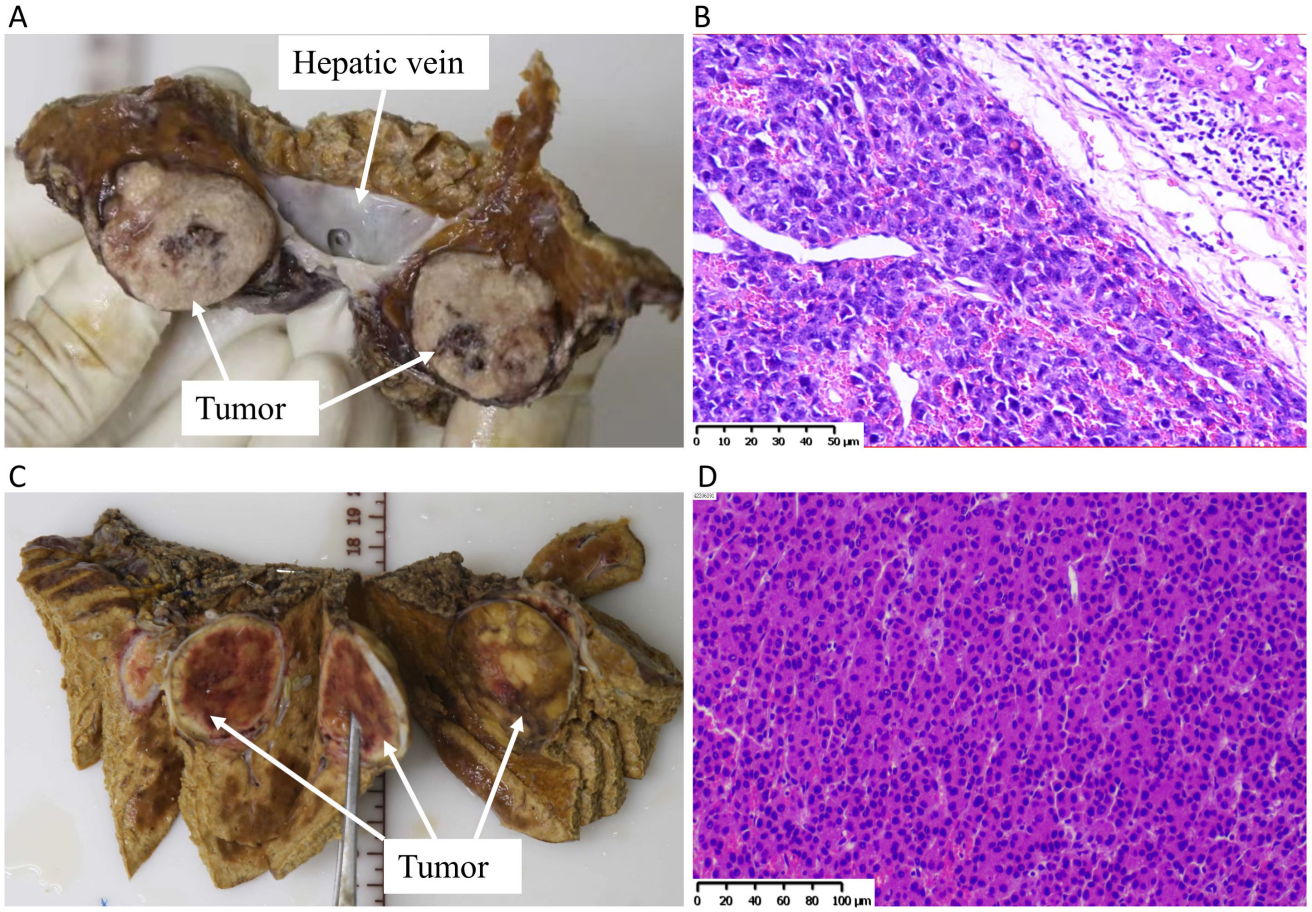
The histopathology of the tumor. **(A)** Anatomical images (longitudinal view of incisal margin and tumor) of excised liver and tumor tissue of case 1. **(B)** H&E-stained section of tumor tissue of case 1; scale = 50 μm. **(C)** Anatomical images (longitudinal view of incisal margin and tumor) of excised liver and tumor tissue of case 2. **(D)** H&E-stained section of tumor tissue of case 2; scale = 100 μm.

Portal vein thrombosis was found on the third postoperative day for case 1, but it did not completely block the portal vein without intervention due to the risk of postoperative hemorrhage, while there were no major surgical complications for case 2. Their liver function recovered well on the seventh postoperative day (**Supplementary Figure S1A**); AFP or des-gamma-carboxy prothrombin of case 1 decreased to its lowest level of 3 months after surgery, while that of case 2 was always at the normal level (**Supplementary Figure S1B**). The clear margins (an R0 margin width ≥0.5 cm) of the tumor were confirmed by *ex vivo* resection under direct vision (**Figures 3B–E**), longitudinal view of excised liver and tumor tissue (envelope integrity, **Figures 4A, C**), and negative pathological incision margin. The patients’ diagnosis was eventually confirmed by pathology as moderately differentiated hepatocellular carcinoma (**Figures 4B, D**). The two patients were discharged on the 28th and 32nd postoperative days. No tumor recurrence was observed during 3 months of follow-up.

## 3 Discussion

Complete surgical resection of tumor tissue with clear margins is considered the most optimal treatment of HCC; unfortunately, only 27%–32.8% of HCC patients are qualified for curative surgery (4). High rates of recurrence or metastasis and poor intraoperative exposure can lead to low resection rates of HCC (5, 6); in turn, poor intraoperative exposure can lead to narrow surgical margins or non-anatomical hepatectomy, which are closely associated with postoperative recurrence of HCC (7). With the rapid progress of new surgical techniques and artificial blood vessel material, the resectability of liver tumors has increased these years. Compared with conventional *in vivo* partial orthotopic hepatectomy, the new surgical technique that combines *ex vivo* surgery and autotransplantation technique is a promising radical resection method to overcome those abovementioned disadvantages (2, 8–11), but there are few reports about the application of *in vivo* total or partial hepatectomy followed by *ex vivo* tumor resection and autotransplantation (3). All the data are from case reports, and almost all the reports carried out a procedure that removed the whole liver to conduct the *ex vivo* tumor resection and autotransplantation, which could lead to the risk of reconstruction of the IVC, unstable intraoperative hemodynamics, and cold ischemia–reperfusion injury of the normal left liver (3, 4). In addition, in 1999, Mukaiya et al. reported the first case of partial liver resection and remnant liver autotransplantation in advanced hepatocellular carcinoma; there are at least four cases of partial liver resection and liver autotransplantation published previously. However, the patients in these case reports were not described as having cirrhosis or did not have a background of cirrhosis, whereas both of our patients had a significant background of cirrhosis (including macroscopic pathology observed during surgery and histopathology). Compared to previous literature, we performed a modified technique of *in vivo* partial hepatectomy followed by *ex vivo* tumor resection and partial liver autotransplantation under the context of shortage and expense of organs, cirrhosis, and insufficient residual liver volume to maximally avoid mass blood loss, narrow the margins, and avoid cold ischemia–reperfusion injury and risk of liver failure. Actually, none of the two cases suffered from severe liver injury or liver failure. More importantly, we did not even observe a short-term recurrence of the tumor, and no patients died. Therefore, for experienced treatment centers, this surgical procedure has certain safety and feasibility in the treatment of locally advanced IVTA liver tumors.

Vascular reconstruction is one of the most crucial steps and most frequent for complex liver tumor resection (12–16). However, artificial vascular grafts are most widely used in these cases (3, 4, 8, 15, 17, 18), with the complications of graft infection, graft patency, and luminal thrombus formation (17–19). In addition, patients require long-term anticoagulation with oral warfarin to maintain long-term patency (18). Here, we used pre-frozen allograft vessels of the vena cava inferior and iliac artery from brain-dead donors to reconstruct the portal vein and outflow of the remaining liver. On the one hand, we directly used vascular materials that fully matched human physiological characteristics, making the anastomosis of blood vessels easier and simpler and avoiding long-term oral warfarin after surgery. On the other hand, we used the toughness and strength of the arteries to maintain the long-term patency of the outflow to avoid the obstruction caused by the narrowing of the outflow or the collapse of the vein material and the compression after liver regeneration. Portal vein thrombosis was found in one case but without bleeding, graft infection, and vessel obstruction observed. The outflow of the two patients had good patency during the follow-up period.

## 4 Conclusion

Our results indicate that *in vivo* partial hepatectomy followed by *ex vivo* tumor resection and partial liver autotransplantation may be a promising option and relatively safe for cirrhotic patients to achieve radical tumor resection, and the use of the pre-frozen donor’s allograft vessels may be a useful vascular reconstruction method. The most important disadvantages of this technique include the surgical complexities and risk of cancer recurrence of the remaining cirrhotic liver.

## Data Availability

The datasets presented in this study can be found in online repositories. The names of the repository/repositories and accession number(s) can be found in the article/**Supplementary Material**.

## Supplementary material

Supplementary Figure 1 Liver function and tumor markers of the two patients.
