# Supplementary material for: Case report: Outflow reconstruction with pre-frozen allograft blood vessels during in vivo partial hepatectomy followed by ex vivo tumor resection and partial liver autotransplantation for locally advanced hepatocellular carcinoma with background of cirrhosis
Source: Front Oncol. 2024 Dec 13;14:1432274. doi: 10.3389/fonc.2024.1432274 (PMC11671509; doi:10.3389/fonc.2024.1432274)
Supplement: Supplementary file 1 [file Table1.docx]

Supplementary Table 1 Abnormal laboratory results of the two patients

| **Examination items** | **Case 1** | **Case 2** |
| --- | --- | --- |
| **Blood route** |  |  |
| WBC (~10^9^/L) | 8.96 | 3.10 |
| HGB (g/L) | 159.00 | 135.00 |
| PLT (~10^9^/L) | 257.00 | 100.00 |
| **Liver function** |  |  |
| TBil (μmol/L) | 6.0 | 20.6 |
| **Des-gamma-carboxy prothrombin (mAU/ml)** | 148.72 | 15.9 |
| **AFP (ng/ml)** | 3752 | 2.3 |
| **ICG R15 (%)** | 1.1 | 4.5 |
| **CT scan of abdomen** | | |
| Tumor number | 1 | 1 |
| Position | S8 | S5/8 |
| Size (cm) | 2.4*2.3*2.2 | 5.1*4.2*4.3 |
| Lymph nodes or distant metastasis | None | None |
| Liver volume/ Residual liver volume（ml） | 1433/363 | 1025/360 |
| **GRBW (%)** | 0.59 | 0.58 |
| Lymph nodes or distant metastasis | None | None |
| Operation time (min) | 915 | 767 |
| Intraoperative blood loss/ blood transfusion (ml) | 100 | 950 |
| Hospitalization time (days) | 35 | 68 |
| **Postoperative complication** |  | |
| Bleeding | None | None |
| Infection | None | Yes（Lungs and abdomen） |
| Bile leakage | None | Yes |
| Thrombosis | None | Portal thrombosis |
| Follow-up (months) | 3 | 3 |
| Tumor recurrence | None | None |

Note: WBC: white blood cell, HGB: hemoglobin, PLT: platelet, TBil: Total bilirubin, ICG R15: The retention rate of indocyanine green at 15 min.
