# Supplementary figures and images for: Case report: Outflow reconstruction with pre-frozen allograft blood vessels during in vivo partial hepatectomy followed by ex vivo tumor resection and partial liver autotransplantation for locally advanced hepatocellular carcinoma with background of cirrhosis
Source: Front Oncol. 2024 Dec 13;14:1432274. doi: 10.3389/fonc.2024.1432274 (PMC11671509; doi:10.3389/fonc.2024.1432274)

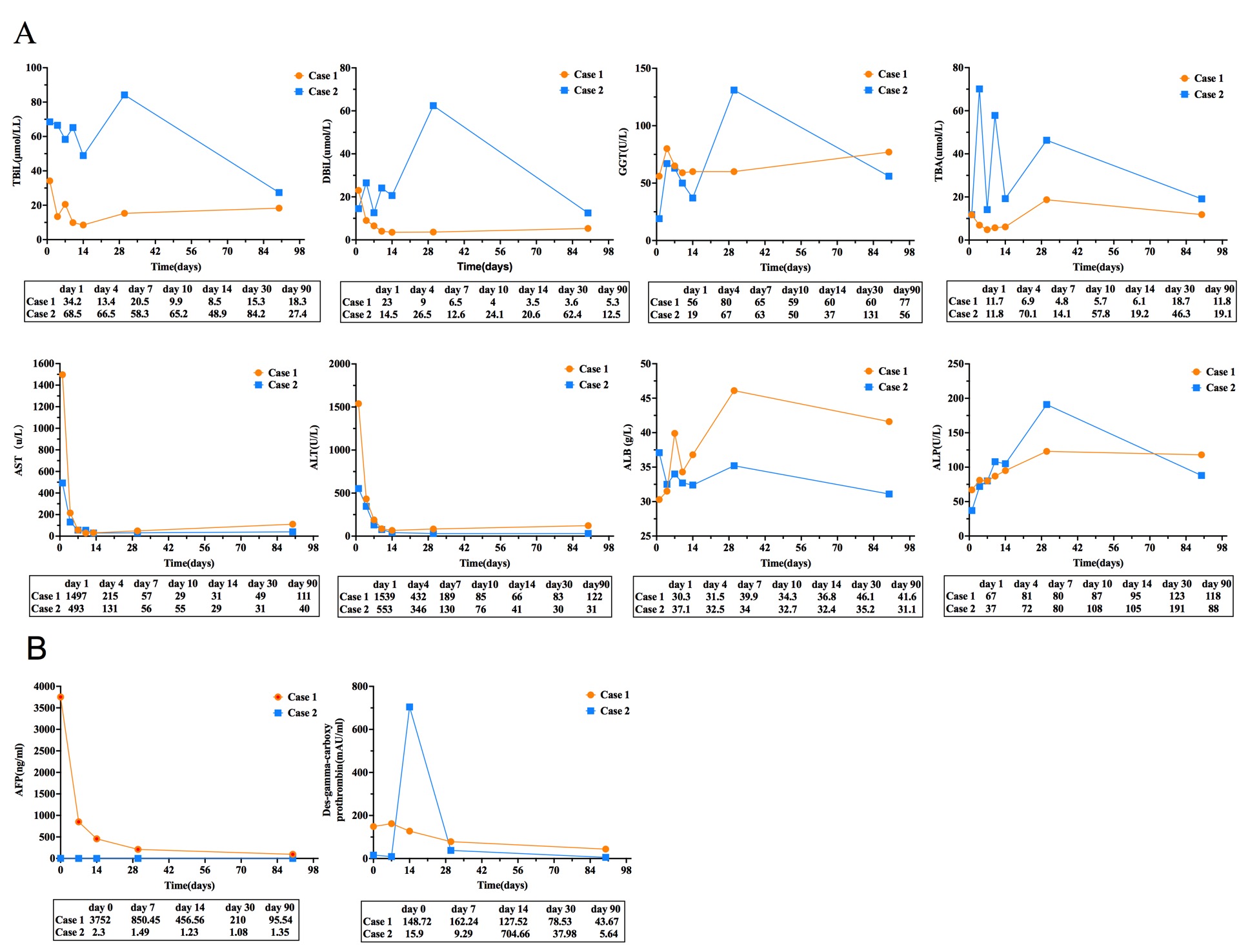

Supplement: Supplementary Figure 1 — Liver function and tumor markers of the two patients. [file Image1.jpeg]
